# How past trauma impacts emotional intelligence: Examining the connection

**DOI:** 10.3389/fpsyg.2023.1067509

**Published:** 2023-05-18

**Authors:** Ryan K. Gottfredson, William J. Becker

**Affiliations:** ^1^Department of Management, College of Business and Economics, California State University, Fullerton, CA, United States; ^2^Department of Management, Pamplin College of Business, Virginia Tech, Blacksburg, VA, United States

**Keywords:** emotional intelligence, neuroscience, trauma, dissociation, hypervigilance, emotional regulation, psychological trauma

## Abstract

Backed by both research and practice, the organizational psychology field has come to value emotional intelligence (EI) as being vital for leader and employee effectiveness. While this field values EI, it has paid little attention to the antecedents of emotional intelligence, leaving the EI domain without clarity on (1) why EI might vary across individuals, and (2) how to best develop EI. In this article, we rely on neuroscience and psychology research to make the case that past psychological trauma impacts later EI capabilities. Specifically, we present evidence that psychological trauma impairs the brain areas and functions that support EI. Establishing psychological trauma has valuable theoretical and practical implications that include providing an explanation of why EI might vary across individuals and providing a focus for improving EI: healing from past trauma. Further theoretical and practical implications for the field of organizational psychology are provided.

## 1. Introduction

Emotional intelligence (EI) is commonly defined as the set of abilities (automatic and deliberate; verbal and nonverbal) that enable a person to generate, recognize, express, understand, and manage their own and others, emotions in order to guide thinking and action that successfully cope with environmental demands and pressures (Van Rooy and Viswesvaran, 2004). Decades of EI research have focused on understanding what EI is, its importance relative to other personal abilities (e.g., cognitive intelligence), the various dimensions and abilities associated with EI, and how to develop the skills associated with EI. Across this research, EI has been hailed and found to be a vital ability for leader and employee effectiveness (Goleman, 1995; Goleman et al., 2002; O’Boyle et al., 2011; McCleskey, 2014).

Despite the value and emphasis organizational scholars have placed on EI, there have been relatively few studies focused on the antecedents of EI (Gunkel et al., 2014). This dearth of research leaves the field of EI without clear answers to two important questions. The first question is: Why do some people possess higher levels of EI than others? Thus far, scholars have found potential antecedents to include Big Five Personality (Johnson et al., 2009), mindfulness (Schutte and Malouff, 2011), and self-determination (Perreault et al., 2014). The answers to this first question are not only limited, but they do not provide much clarity for the second question, which is: How does one best develop EI? For example, it seems unlikely that one can change their personality as a means of improving EI.

While we are open to multiple explanations for (1) why some people possess higher versus lower EI and (2) how to best develop EI, we want to explore one novel and compelling explanation that can potentially answer both questions. This explanation involves an investigation into the neuroscience and psychological research on psychological trauma and how such trauma predictably leads to neural adaptations that hinder EI and EI abilities. Connecting psychological trauma to EI not only has the potential to explain why some people have lower EI than others (e.g., they have experienced more psychological trauma), but it also has the potential to explain at least one method of helping people increase their EI (e.g., healing from past trauma).

We believe that shedding light on the connection between psychological trauma and EI not only addresses important theoretical questions but it lays out clear, practical implications for improving EI. And, to add magnitude to these implications, it is important to acknowledge that psychological trauma is not a rare phenomenon. Psychological trauma researchers and experts estimate that over 70% of adults have experienced significant psychological trauma in their lives (Benjet et al., 2016; National Council for Behavioral Health, 2023). Thus, if psychological trauma is common and impacts later EI capabilities, and EI is valuable for leader and employee effectiveness, we are touching on a connection that deserves greater research attention.

## 2. What is emotional intelligence?

There are two major models of EI: ability EI and trait EI. Decades of research trying to distinguish these models from each other have led scholars to acknowledge that these are different constructs with independent literatures (Brannick et al., 2009; Petrides, 2011).

Ability EI is largely viewed as a broad ability involved in the cognitive processing of emotions and emotional information (Mayer et al., 2004; Elfenbein and MacCann, 2017). Ability EI is a perspective on EI that (1) suggests that EI is more similar to cognitive intelligence than personality, (2) implies that EI is something that can be developed similar to other cognitive processing abilities, and (3) is measured through maximum-performance tests (Van Rooy et al., 2005; Petrides et al., 2007a; Joseph and Newman, 2010). Being a “broad ability,” ability EI comprises a variety of emotional abilities, which are most commonly recognized to include emotion perception, emotion facilitation of thought, emotion understanding, and emotion regulation (e.g., Mayer et al., 2016), although there are others who identify other emotion-related abilities that may or should be connected to the “broad ability” of EI (e.g., Elfenbein and MacCann, 2017).

Trait EI is considered a constellation of emotional self-perceptions located at the lower levels of personality hierarchies (Petrides, 2010). Trait EI is a perspective on EI that (1) suggests that EI is more similar to personality than cognitive intelligence; (2) implies that EI is something that is not very easy to develop, similar to other personality abilities (e.g., Big Five Personality traits); and (3) is measured through self-report questionnaires (Petrides and Furnham, 2001; Van Rooy et al., 2005; Petrides et al., 2007a,b; Joseph and Newman, 2010). Trait EI also comprises a variety of emotion-related traits, with emotional self-efficacy being acknowledged most commonly (Petrides et al., 2007a; Petrides, 2010, 2011).

For the purposes of this paper, we focus on ability EI for two primary reasons. First, we are interested in providing an explanation for how people can improve their EI, and the ability EI perspective is more skill-based, as opposed to trait-based, theoretically suggesting that it is something that can be improved. Second, while there is a substantial amount of research that focuses on trait EI, when neuroscience researchers study EI, they primarily use ability EI measures of EI. As we will be reporting on the neural nature of EI stemming from neuroscience research, it is important our perspective is aligned with this prior research. Henceforth, when we refer to EI, we are referring specifically to ability EI.

## 3. The nature of emotional intelligence

Since EI is a personal attribute that can be developed and comprised of a number of different abilities (e.g., emotion perception, emotion facilitation of thought, emotion understanding, and emotion regulation), EI is considered to be a skill-based ability (e.g., Caruso et al., 2002; Salovey and Grewal, 2005; Mayer et al., 2008). When seen as a skill-based ability, EI deficiencies are seen as skill deficiencies, with the implication that the improvement of EI comes through common skill development practices including the gaining of knowledge and the practice of skills.

While the skill-based view of EI has merit (see Kotsou et al., 2019), advances in neuroscience and psychology research focused either directly on EI or common EI-related abilities identifies (1) specific brain areas, networks, and functionality that underlie EI abilities (e.g., Hogeveen et al., 2016; Andrewes and Jenkins, 2019); (2) some factors that negatively affect the EI-related brain areas, networks, and functionality, leading to lower or reduced EI (e.g., Andrewes and Jenkins, 2019; Leroy et al., 2022); and (3) what can be done at a neural level to heal and improve these brain areas and systems, thus leading to improved neural capabilities for EI (e.g., Malejko et al., 2017). This research suggests that there may be theoretical and practical value in considering EI to be a neural-based ability. Theoretically, viewing EI as a neural-based ability may allow EI researchers to consider antecedents of EI that they may not consider otherwise. Practically, it may allow EI researchers to consider different developmental practices that are different in nature from the standard skill development-related practices.

The neural perspective of EI stems from research focused on understanding the parts and functionality of the brain through the study of brain injuries. Neuroscience scholars have long been interested in understanding (1) the different parts of the brain and what they are responsible for and (2) how the different parts of the brain function together and influence how individuals function and operate. One method to shed light on brain regions and functionality is to compare people who have had physical damage to a certain brain region as a result of stroke, surgery, or injury (commonly referred to as lesions) to normal healthy adults.

Below, we will summarize what brain lesion research has taught us about the brain areas and brain networks that seem to play important roles in EI and EI-related abilities. But, before summarizing that research, we first provide an overview of the brain areas and networks that have been identified as playing a role in EI and EI-related abilities.

## 4. Brain areas and networks involved in emotional intelligence

Neuroscience researchers who are interested in studying how people recognize and respond to salient events in our environment (e.g., including emotions within oneself or the emotions of others) have found that there are three distinct higher-order functional networks in the brain that work together to foundationally influence what individuals recognize and how they respond to the salient events they encounter (Sridharan et al., 2008; Menon, 2013). They are the salience network (SN), the default mode network (DMN), and the central executive network (CEN).

The SN consists of key limbic and prefrontal regions of the brain that include the ventrolateral prefrontal cortex, anterior insula, amygdala, and anterior cingulate cortex (Menon and Uddin, 2010; Yuen et al., 2014). It is responsible for the selection, segregation, and interpretation of stimuli as well as the switching between the DMN and CEN (Sridharan et al., 2008; Goulden et al., 2014).

The DMN consists largely of the ventromedial prefrontal cortex and the posterior cingulate cortex, and is activated and primarily operates during resting states (Menon and Uddin, 2010; Yuen et al., 2014). It runs individuals’ non-conscious automatic processing and has been found to support self-referential activities (e.g., interoception, autobiographical memory retrieval, imagining the future) as well as high-level social cognitive tasks (Sridharan et al., 2008; Goulden et al., 2014).

The CEN consists largely of the dorsolateral prefrontal cortex and posterior parietal cortex (Menon and Uddin, 2010; Yuen et al., 2014). It is responsible for the active and conscious maintenance and manipulation of information in working memory, and for judgment and decision making in the context of goal-directed behavior (Sridharan et al., 2008; Goulden et al., 2014).

Table 1 presents the objective of each network and the areas of the brain involved in each network (Sridharan et al., 2008; Menon and Uddin, 2010; Goulden et al., 2014; Yuen et al., 2014).

**Table 1:** The networks and areas of the brain involved in EI abilities.

Network	Network purposes	Key areas of brain involved
Central executive network (CEN)	Responsible for the active maintenance and manipulation of information in working memory, and for judgment and decision making in the context of goal directed behavior.	Dorsolateral prefrontal cortex – Plays a key role in the executive functions of working memory, cognitive flexibility, planning, inhibition, and abstract reasoning.
		Posterior parietal cortex – Plays an important role in planned movements, special reasoning, and attention.
Default mode network (DMN)	Activated during resting states, but has been found to support self-referential activities (e.g., interoception, autobiographical memory retrieval, imagining future) as well as high-level social cognitive tasks.	Ventromedial prefrontal cortex – Plays an important role in the processing of risk and fear. It regulates the amygdala, and thus plays an important role in the inhibition of emotional responses and in the process of decision-making and self-control.
		Posterior cingulate cortex – Plays a role in pain and episodic memory retrieval, including the emotional salience involved in such memory retrieval.
Salience network (SN)	1. Responsible for the selection, segregation, and interpretation of stimuli. 2. Drives the switching between DMN and CEN.	Ventrolateral prefrontal cortex – Plays an important role in response inhibition and goal-appropriate response selection.
		Anterior insula – Plays an important role in the awareness and control of emotions as well effective navigation of social experiences.
		Amygdala – Plays an important role in the processing of memory, decision making, and emotional responses (including fear, anxiety, and aggression).
		Anterior cingulate cortex – Plays an important role in attention allocation, reward anticipation, decision making, ethics, impulse control, and emotion.

## 5. Brain lesion research related to emotional intelligence

By comparing healthy brains to brains with lesions, neuroscientists have consistently found that when certain areas of the brain have been damaged, those individuals have reduced EI and EI-related abilities. The following presents some of the key findings associated with what brain areas and networks are connected to EI and EI-related abilities from brain lesion research.

### 5.1. Brain areas linked to emotional intelligence and emotional intelligence-related abilities

Specifically, neuroscience researchers have found that the SN, and its areas (e.g., amygdala, anterior inula), is the primary hub for the feeling, awareness, and processing of emotions, as it is in charge of automatic emotional attention (Smith et al., 2018). As such, the health and optimal functionality of the SN and its areas have been identified as being foundational for EI and EI-related abilities. Findings to support the SN’s role in EI include the following: People with physical damage to the brain areas within the SN have a diminished ability to detect fearful and other negative facial expressions (Adolphs et al., 2002; Santos et al., 2016), recognize dramatic music intended to evoke fear (Gosselin et al., 2005), experience the feelings of fear (Feinstein et al., 2011), be reliable and reputable partners (Tranel and Hyman, 1990), extend the appropriate level of trust to others (Belfi et al., 2015), and demonstrate empathy and control aggression (Sterzer et al., 2007; Gu et al., 2012).

Neuroscience researchers have also found that the DMN plays a strong role in the interpretation, recognition, and regulation of emotion (Sawaya et al., 2015; Killgore et al., 2017; Smith et al., 2018). For example, neuroscience researchers have found that people who have experienced physical damage to the brain areas within the DMN have diminished ability to do the following, which are indicative of low EI: emotionally regulate oneself (Hornak et al., 2003), engage in appropriate social behavior (Damasio and Anderson, 1993), be self-aware (Hornak et al., 2003), engage in self-sight or self-reflection (Koenigs and Grafman, 2009; Philippi and Koenigs, 2014), and be focused (as opposed to reactive; Andrewes and Jenkins, 2019).

The CEN, while necessary for many things, appears to not play as dominant of a role with EI and EI-related abilities. Research has indicated that the CEN plays a role in the interpreting of emotions (Pan et al., 2014; Bajaj and Killgore, 2021), but most of the neuroscientific research investigating the role the three brain networks play in emotional intelligence generally either (1) only considered the connectivity and involvement between the DMN and SN (and not CEN and SN; e.g., Takeuchi et al., 2013; Killgore et al., 2017), or (2) indicated greater connectivity and involvement between the DMN and SN than the CEN and SN (for examples, see Smith et al., 2018; Li et al., 2021). In fact, Smith et al. (2018) suggest that the CEN may play a more indirect role in EI by being responsible for controlling goal-related attention and cognition, which may impact how the brain uses emotions (e.g., suppression or amplification).

### 5.2. Brain network functionality linked to emotional intelligence and emotional intelligence-related abilities

Lesion studies have found that when there is physical damage to the brain areas of one network, there is a disruption to how the different brain networks work together, which collectively impacts individuals’ EI and EI-related abilities. For example, lesion studies have also found that when there is physical damage to the brain areas within the DMN, there is reduced regulation of the amygdala, evidence of diminished brain network functionality between the DMN and SN (Van der Horn et al., 2016a,b; Andrewes and Jenkins, 2019). The consequence of this has been found to include excessive and dysregulated emotional reactivity (Van der Horn et al., 2016a,b; Jenkins et al., 2018).

### 5.3. Summary of brain lesion research related to emotional intelligence

To summarize the lesion research related to EI, Hogeveen et al. (2016) reviewed past research on how brain injuries might affect individuals’ EI, providing ample evidence that brain injuries reduce individuals’ abilities to possess five EI-related abilities (emotional awareness, emotional recognition, emotional regulation, affective empathy, and theory of mind). Altogether, brain lesion research provides ample evidence that there is a strong neural component to EI and EI-related abilities, suggesting that EI is foundationally a neural-based skill.

When we acknowledge that EI is a neural-based skill within the context of lesion studies, two sets of questions are worth considering. The initial question in the first set of questions is: How frequently do people experience brain injuries to the extent that it negatively impacts their EI? While we do not have a concrete answer to this, the Centers for Disease Control and Prevention (2019) report that approximately 17 people per 100,000 in the United States have a traumatic brain injury on an annual basis. From this, we can infer that (thankfully) relatively few experience brain injuries leading to reduced capacity for EI. The next question involves whether the effects of lesions on EI can be reversed. All evidence suggests that the answer is “it depends.” The nature of the lesion (e.g., stroke, surgery, injury) and the severity of the lesion play primary roles (Ponsford et al., 2008; Kreutzer et al., 2016).

The second set of questions looks beyond lesions. First, are there other things that could negatively affect the brain areas and functionality necessary for EI, which might explain why some people have high EI and others have low EI? If there is, the second and third questions are: Are they common? And, can the negative effects on EI be reversed? In the next section, we provide answers to these questions.

## 6. Exploring the connection between psychological trauma and emotional intelligence

The fields of neuroscience and psychology have identified psychological trauma as negatively affecting the brain areas and functionality necessary for EI. In this section, we define psychological trauma, report the evidence that it impacts the brain areas and functionality necessary for EI, and examine research that investigates if there are means by which the negative effects of psychological trauma on the brain areas and networks involved in EI can be reversed.

### 6.1. What is psychological trauma?

Psychological trauma is an individual’s experience of a negative event or enduring condition where the individual’s immediate ability to process his/her emotional experience is overwhelmed (Pop-Jordanova, 2022). In fact, the term “trauma” connotes the idea of being psychologically wounded (Schimmenti, 2018). Psychological trauma can occur at any life stage and in any setting. There is ample research on childhood trauma, often focusing on abuse or neglect (Mandelli et al., 2015; Malarbi et al., 2017; Humphreys et al., 2020); trauma related to military service (Kitchiner et al., 2019; Straud et al., 2019); and traumatic work events in corporate settings (Stergiopoulos et al., 2011; Marin et al., 2019; Pihl-Thingvad et al., 2019). For this article, we are not concerned about the type of trauma or when it occurs in one’s life. Instead, we seek to highlight neuroscientific evidence demonstrating that trauma causes acute and predictable changes in the brain that can inhibit EI abilities.

Unlike brain lesions, which are rare, psychological trauma is incredibly common. Experts estimate that over 70% of adults have experienced significant trauma in their lives (Benjet et al., 2016; National Council for Behavioral Health, 2023), and given the observed neural changes that occur in individuals who experience trauma, which we detail below, we believe that we are addressing a topic that affects a large proportion of leaders and employees. Therefore, we believe that this is an important topic that affects many people at a personal level and almost all people at a relationship level.

### 6.2. The effect of psychological trauma on brain areas

Within the body of neuroscientific research related to the impact of trauma on the brain, a majority of the extant research focuses on how psychological trauma impacts the SN brain areas and the network functionality between the SN and the DMN or CEN (Tursich et al., 2015). There is little research that focuses on the impact of psychological trauma on solely the DMN or CEN. Thus, in this section, we summarize the neuroscientific research on the effect of psychological trauma and the SN brain areas. Then, in the next section, we summarize the neuroscientific research related to the effect of psychological trauma on brain network functionality.

Neuroscientific research has found that psychological trauma alters the SN brain areas in a number of different ways. First, several studies show that past psychological trauma is associated with reduced volume in the SN brain areas (Weniger et al., 2009; Aas et al., 2012; Herringa et al., 2012; Veer et al., 2015; Weissman et al., 2020; Nogovitsyn et al., 2022). Second, psychological trauma is also thought to contribute to heightened response and overactivity in SN brain areas (Fonzo et al., 2010; Grant et al., 2011; Yehuda et al., 2015; Kleshchova et al., 2019; Duval et al., 2020). Third, psychological trauma seems to reduce functional connectivity within the SN brain areas (Nooner et al., 2013; Thomason et al., 2015; Zhang et al., 2016). Altogether, these trauma-induced alterations to the SN brain areas have been demonstrated to reduce individuals’ ability for cognitive control and emotional processing (Aas et al., 2012; Thomason et al., 2015; Leroy et al., 2022) and hinder one’s ability to respond to the emotions of others in appropriate ways (Kim et al., 2014).

### 6.3. The effect of psychological trauma on brain functionality

From a brain network perspective, neuroscientific research consistently finds that the brain responds to psychological trauma with one of two neural adaptations that can impact post-trauma brain network functionality and associated EI abilities. These adaptations are hypervigilance and dissociation. These adaptations are commonly seen as responses meant for self-preservation against future trauma, but can be inhibiting in circumstances not involving threats of potential trauma (Van der Kolk, 2002). Paradoxically, the brain adaptations that lead to hypervigilance are seemingly opposite to the brain adaptations that lead to dissociation (Nardo et al., 2013). Thus, each adaptation produces quite different patterns of emotional response. Yet, both tend to limit EI abilities.

#### 6.3.1. Primary consequence of psychological trauma: hypervigilance

Repeatedly, trauma researchers find that one consequence of psychological trauma is an overactive SN and a DMN that struggles to regulate the overactivity of the SN (Williams et al., 2006; Felmingham et al., 2007; Admon et al., 2013; Wang et al., 2013; Rabellino et al., 2018). The outcome of this particular neural adaptation is hypervigilance. Hypervigilance is the state of being “carefully watchful for possible danger or difficulties to an excessive degree,” which leads to “a lack of normal integration of thoughts, feelings, and experiences into the stream of consciousness and memory” (Bernstein and Putnam, 1986, p. 727). When people are hypervigilant, their overactive SN overrides regulation by the DMN and makes them prone to interpret stimuli that are usually considered safe as being unsafe (Kimble et al., 2013; Yoon and Weierich, 2016). As a result, they are prone to suspicion, mistrust, and carrying negative expectancies for the future; and they overly engage in safety-seeking thinking and behaviors (e.g., planning escape routes, avoid feeling trapped). For those who have experienced psychological trauma, the neural changes that lead to hypervigilance are a natural defense mechanism to help limit future exposure to psychological trauma. While hypervigilance may be appropriate and adaptive in potentially traumatic situations, it is largely dysfunctional outside of potentially traumatic situations (Kleshchova et al., 2019).

When individuals are hypervigilant, they are continuously on high alert for potential threats. This self-protection reduces their positive emotions and diminishes their ability to recognize, understand, and evaluate others’ emotions. Additionally, this hypervigilance means that they, compared to individuals with normal levels of vigilance, are prone to struggle to control and regulate their emotions appropriately. Effectively, they will have a narrower window of tolerance that throws their body into fight, flight, or freeze mode, all conditions where executive functioning and EI abilities are inhibited (Corrigan et al., 2011). The dysregulated arousal in the SN as the result of psychological trauma leads to distractibility, inability to disengage from distracted thinking, numbing, avoidance, poor recovery from challenges, and exhaustion, among others (Kimble et al., 2013; Yoon and Weierich, 2016; Kleshchova et al., 2019). In all, hypervigilance inhibits one’s ability to perform the core functions of EI: (1) be cognizant of their and others’ emotions, and (2) navigate their or others’ emotions effectively, perhaps especially in the instances these individuals need EI the most (e.g., stressful, but not inherently unsafe situations).

#### 6.3.2. Primary consequence of psychological trauma: dissociation

Another common consequence of psychological trauma is a different neural adaptation labeled dissociation. Dissociation involves brain functioning that is essentially the opposite of hypervigilance, in that the DMN is overactive and overregulates the SN (Moser et al., 2013; Nicholson et al., 2017; Rabellino et al., 2018; Fani et al., 2019). Dissociation is a protection mechanism in the moment of trauma that results in the individual cognitively and emotionally detaching themselves from the traumatic experience (Dalenberg et al., 2012; Schimmenti and Caretti, 2014; Schimmenti, 2018). While this emotional detachment may help the individual survive and cope with the traumatic experience, it has an overall long-term dampening effect on one’s ability for emotional self-awareness thereafter.

Dissociation researchers have found that when the DMN overregulates the SN, there is a suppression of the things that the SN is normally responsible for, including emotion, perception, body representation, motor control, and behavior (Dalenberg et al., 2012; Lebois et al., 2021). The overarching consequence of this suppression is a decreased capacity to connect with both the emotional and physical sensations in their body (Ozdemir et al., 2015), which results in a variety of impairments including decreased sensitivity to salient cues, reduced interoceptive abilities, depersonalization, body-ownership distortions, and sensory alterations (Rabellino et al., 2018; Fani et al., 2019).

Thus, the DMN’s suppression of the SN not only dampens one’s ability to be aware of and recognize their own emotions (Fani et al., 2019), but because they have decreased sensitivity to salient cues, they also have an impaired ability to recognize and respond appropriately to the emotions of others (Moser et al., 2013). Together, research on dissociation implies that those who are dissociated have a diminished capacity for the brain network functioning necessary for EI (e.g., Craparo, 2014; Ozdemir et al., 2015).

#### 6.3.3. Summary of the effects of psychological trauma on emotional intelligence

In this section, we present research that demonstrates that psychological trauma causes neural adaptations that hinder individuals’ EI abilities. In essence, the effects of psychological trauma on EI appear to be similar to the effects of one having a brain injury or lesion within the SN network or DMN on EI. While there is more sensitivity around the prevention of brain injuries than ever before (consider the National Football League’s increasing sensitivity around concussions and chronic traumatic encephalopathy (CTE)), the connection we have made between psychological trauma and EI suggests there should be increased sensitivity of psychological trauma as well, particularly given how common psychological trauma is relative to brain lesions.

After considering the research demonstrating that psychological trauma negatively affects brain areas and functionality necessary for EI, and given the estimates that over 70% of adults have experienced psychological trauma in their lives, we believe that we have identified an explanation for why some people have higher EI than others. While this answers an important theoretical question, we next try to answer a more practical question: Can the negative neural effects of psychological trauma on EI be reversed?

### 6.4. Reversing the effects of psychological trauma on emotional intelligence: healing the mind

We recognize that there are physical limitations associated with healing the mind in the case of physical damage (i.e., lesions). But, in the case of neural adaptations related to psychological trauma, neuroscience is thus far indicating that it is possible for healing in the brain areas and network functionality involved in EI. Although, we believe that more research is needed in this space to answer this question more conclusively as well as provide greater direction on the best approaches to heal the mind for greater EI.

Neuroscience research focused on healing psychological trauma is relatively nascent, but growing. In a review of current trauma therapy modalities across 19 studies, including the modalities of cognitive behavioral therapy, eye movement desensitization and reprocessing, cognitive therapy, exposure therapy, and mindfulness-based intervention, Malejko et al. (2017) concluded that therapy has repeatedly been shown to decrease the activity in the limbic brain regions (primarily SN) and increase the activity in the frontal brain areas (including the DMN), a seeming reversal of hypervigilance.

Studies published after the Malejko et al. (2017) review are continuing to find similar outcomes, sometimes including additional therapies (e.g., psychedelics; Zhu et al., 2018; Santarnecchi et al., 2019; Mertens et al., 2020; Bryant et al., 2021). In one notable study, Takamiya et al. (2018) found that electroconvulsive therapy, an effective treatment for depression, increased brain volume in the limbic structures (primarily SN), suggesting that it may be possible to counteract common structural brain deficiencies that result from psychological trauma.

In another notable study, Leroy et al. (2022) investigated the healing of brain network functionality associated with PTSD patients prone to flashbacks and nightmares (called “re-experiencing trauma”). These researchers identified that these re-experiences are associated with a dysregulation involving a stronger DMN, a more unstable CEN, and an overactive SN (particularly the anterior insula). More importantly, they found that trauma memory reactivation therapy using propranolol (considered a putative reconsolidation blocker) reduced the overactivation of the anterior insula, allowing for better cross-talk between the DMN and CEN, reducing both re-experiencing trauma and dissociative experiences. Vuper et al. (2021) found similar findings in their study involving the treatment of cognitive behavioral therapy.

Overall, there is meaningful evidence suggesting that even when individuals have experienced neurological effects of psychological trauma that diminish EI, there are methods for improving the health of the brain areas and brain network functionality responsible for EI and EI-related abilities. Further, we recognize that post-traumatic growth research suggests that traumatic events, when properly resolved and addressed, can be catalysts for individual growth, change, and resilience (Maitlis, 2020).

## 7. Discussion

There is an accepted understanding that leaders and employees possessing EI and its related abilities (e.g., emotional awareness, emotional recognition, emotional regulation, affective empathy, theory of mind) are more effective than those who possess lower EI and diminished EI abilities. For example, Alonazi (2020) found a positive relationship between leaders’ EI and success and performance during a crisis to the degree that it allows organizations to gain a competitive advantage. Yet, there has been relatively little research into the antecedents of EI. As a result, there is little clarity on (1) what explains why some people have higher versus lower EI, and (2) how one can develop or improve their EI.

In this article, we have sought to introduce psychological trauma as a possible explanation of why ability EI varies across individuals. To do this, we initially leaned on brain lesion studies to identify the brain areas and functionality necessary for EI. Then, we provided research from the neuroscience and psychological domains finding that psychological trauma negatively impacts the brain areas and functionality necessary for EI. While we are open to there being other explanations for variations in EI than brain lesions and psychological trauma, our focus on the connection between psychological trauma and EI has important theoretical and practical implications.

### 7.1. Theoretical implications

In this paper, we have introduced psychological trauma as a possible explanation for variations in EI. There are a variety of theoretical implications for doing so. First, as mentioned previously, there are relatively few studies that have investigated the factors that impact individuals’ EI. We believe we are adding an explanation to this field of study. Second, by initially exploring the neural underpinnings of EI from brain lesion and psychological trauma research, we have provided strong evidence that ability EI is not simply a skill-based ability, rather, it seems to be a neural-based ability, enhancing our understanding of the nature of EI. Third, by identifying EI as a neural-based ability, we open the door for the improvement of the measurement of EI, something that has been called for (see Jordan et al., 2003; Van Rooy and Viswesvaran, 2004; Conte, 2005). Thus far, ability EI is primarily assessed through self-report measures. But, with advances in brain imaging technology, it might be possible to measure EI by directly looking at the health and functionality of the brain parts involved in EI. Fourth, by identifying psychological trauma as an explanation for why individuals have high or low EI, we provide strong theoretical backing for further exploration into how to best heal the impacted brain areas and functionality from psychological trauma as a means of improving EI. Finally, we open the door for broader theorizing associated with leader and employee functioning and performance. To our knowledge, there is little organizational research that investigates the neuroscience associated with leader and employee functioning and performance and/or the role past psychological trauma plays in leader and employee functioning and performance. We believe this article provides some theoretical grounding for such research.

### 7.2. Practical implications

With little clarity on the antecedents of EI, there has been relatively little emphasis on how to improve ability EI in the academic literature. But, there is widespread use of training and development efforts to improve EI in organizational contexts. Our research not only illuminates new ways of improving EI, but also exposes the limitations of common EI development efforts.

Most EI development efforts focus primarily on skill development (Kotsou et al., 2019). But, we have provided evidence that EI has a strong neural component to it. As such, it is valuable to question whether common skill development efforts designed to improve EI improve the neural functioning necessary for EI, because if they do not, then the value of the skill development efforts may be only incrementally helpful, at best.

By identifying psychological trauma as an antecedent of EI, and acknowledging the evidence that the negative effects of psychological trauma on EI can be reversed (see Malejko et al., 2017; Takamiya et al., 2018; Zhu et al., 2018; Santarnecchi et al., 2019; Mertens et al., 2020; Bryant et al., 2021; Vuper et al., 2021; Leroy et al., 2022), EI developers now have a specific focus for the development of EI: helping individuals heal from their past psychological trauma. For organizations, this may mean that if they want to improve their leaders’ and employees’ EI, they may need to provide increased resources and access to therapeutic techniques found to be helpful in the healing of the brain areas and functionality necessary for EI.

### 7.3. Future research

There is ample evidence to support two facts: first, EI is a vital skill for leaders and employees (Goleman, 1995; Goleman et al., 2002; O’Boyle et al., 2011; McCleskey, 2014); and second, psychological trauma is incredibly common, with estimates of over 70% of adults having experienced significant psychological trauma (Benjet et al., 2016; National Council for Behavioral Health, 2023). Relying upon neuroscience and psychology research, we have been able to make a connection between these two facts, identifying psychological trauma as one possible inhibitor of EI. Given the commonness of psychological trauma and the importance of EI, we believe that this article will lead to increased theoretical and practical attention to this connection. Specifically, we hope this article spurs future research to confirm the extent to which psychological trauma affects EI. Also, we hope this article leads to research efforts to provide increased clarity on the techniques and developmental efforts that can help people heal the brain parts and functionality necessary for EI. Finally, by establishing that there are neural underpinnings to a vital ability for leader and employee effectiveness, we hope that our research leads to a broader investigation into the neuroscience behind leadership and employee effectiveness.

### 7.4. Conclusion

A primary focus of EI research has been directed at the consequences of EI, leading to the acknowledgment of EI being a highly valued ability for leader and employee effectiveness. But unfortunately, there has been scant research investigating the antecedents of EI, leaving a lack of clarity for why different individuals have high versus low EI and how to best develop EI. In this article, relying upon neuroscience and psychological research, we introduce psychological trauma as (1) one explanation for why EI varies across people, and (2) an avenue for EI development: healing from psychological trauma. We believe establishing this psychological trauma to EI connection will lead to improved theorizing, practice, and research associated with EI.

## Author contributions

RG conceptualized the paper, conducted the primary literature review, wrote the first draft, and did final edits. WB supplemented the literature review, edited, and revised, strengthened the paper. All authors contributed to the article and approved the submitted version.

## Conflict of interest

The authors declare that the research was conducted in the absence of any commercial or financial relationships that could be construed as a potential conflict of interest.

## Publisher’s note

All claims expressed in this article are solely those of the authors and do not necessarily represent those of their affiliated organizations, or those of the publisher, the editors and the reviewers. Any product that may be evaluated in this article, or claim that may be made by its manufacturer, is not guaranteed or endorsed by the publisher.
